# Amplification Artifact in SARS-CoV-2 Omicron Sequences Carrying P681R Mutation, New York, USA

**DOI:** 10.3201/eid2804.220146

**Published:** 2022-04

**Authors:** Adriana Heguy, Dacia Dimartino, Christian Marier, Paul Zappile, Emily Guzman, Ralf Duerr, Guiqing Wang, Jonathan Plitnick, Alexis Russell, Daryl M. Lamson, Kirsten St. George

**Affiliations:** NYU Langone Health, New York, New York, USA (A. Heguy, D. Dimartino, C. Marier, P. Zappile, E. Guzman);; NYU Grossman School of Medicine, New York (A. Heguy, R. Duerr, G. Wang);; New York State Department of Health, Albany, New York, USA (J. Plitnick, A. Russell, D.M. Lamson, K. St. George);; State University of New York at Albany, Albany (K. St. George)

**Keywords:** COVID-19, 2019 novel coronavirus disease, coronavirus disease, severe acute respiratory syndrome coronavirus 2, SARS-CoV-2, viruses, respiratory infections, zoonoses, genomics, sequence analysis, amplification artifact

## Abstract

Of 379 severe acute respiratory syndrome coronavirus 2 samples collected in New York, USA, we detected 86 Omicron variant sequences containing Delta variant mutation P681R. Probable explanations were co-infection with 2 viruses or contamination/amplification artifact. Repeated library preparation with fewer cycles showed the P681R calls were artifactual. Unusual mutations should be interpreted with caution.

The recently emerged Omicron variant of severe respiratory syndrome coronavirus 2 (SARS-CoV-2) (*1*) is highly transmissible and partially immune evasive (*2*). Omicron contributes to the recent surges in coronavirus disease (COVID-19) case numbers, even in locations with highly vaccinated populations and vaccination requirements for indoor dining and events, such as New York, New York, USA. During our ongoing SARS-CoV-2 genomic surveillance using full-genome sequencing with random sample selection from positive cases at NYU Langone Health, a large metropolitan healthcare system, we observed a rapid rise in Omicron and displacement of Delta. We analyzed a subset of 379 Omicron sequences, which we deposited in GISAID (https://www.gisaid.org), all BA.1 sublineage, from cases detected during November 30, 2021–January 5, 2022.

Detailed methods were recently described (*3*). In brief, we used the xGen SARS-CoV-2 Amp Panel 96rxn (Integrated DNA Technologies [IDT], https://eu.idtdna.com) and 18 or 24 cycles for the multiplex PCR step of library amplification. Of the 379 samples, amplified using 24 cycles regardless of cycle threshold (C_t_), we detected 86 with P681R, a key Delta mutation associated with increased transmissibility, fusogenicity, and pathogenicity (*4*), distinct from the P681H mutation of Omicron. The presence of P681R in Omicron was cause for concern because it could be associated with higher pathogenicity/transmissibility.

Closer examination indicated that the Omicron sequences with P681R contained varying numbers of P681H reads (median frequency of P681R call, a G at nucleotide position 23604, was 0.79 [range 0.43–0.98]). This observation could indicate either co-infection with Omicron and Delta or contamination/artifact of sample processing and library preparation. We also observed that sequences with P681R were from samples with higher C_t_s according to real-time detection assays compared with those with P681H, (open reading frame, TaqPath COVID-19 Combo Kit, Applied Biosystems, https://www.thermofisher.com) (median C_t_ = 22 for P681H and 28 for P681R; p<7.0803 × 10^–37^). We also observed that coverage of nt 23604 was higher when the call was G (Delta context) than when the call was A (Omicron context) (Figure). These observations are consistent with contamination of Omicron samples with lower C_t_ by Delta sequences, possibly exacerbated by overamplification and preference of the polymerase for the specific amplicon flanked by primers at positions 23534 and 23641 (covid19 genome_200–29703_s20720_D_32 in the IDT xGen kit).

**Figure:**
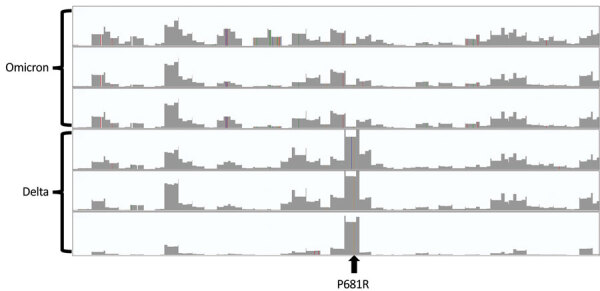
Integrated Genome Viewer (https://software.broadinstitute.org) plots of sequencing coverage of the entire length of the spike gene (3,821 nt, x-axis) of 3 representative sequences of the Omicron variant and 3 representative sequences of the Delta variant of severe acute respiratory syndrome coronavirus 2, processed in the same batch. The y-axis for each sequence is 250,000 reads. Although coverage plots are basically identical for other regions of the spike gene, coverage of the P681 position is the highest for Delta and one of the lowest for Omicron.

To investigate further, we repeated the library preparation and sequencing on 13 random samples previously assigned as P681R and 13 assigned as P681H as controls and changed 2 parameters: reverse transcriptase (RT) and PCR cycles. We prepared 10 samples with SuperScript IV Reverse RT (recommended by IDT xGen kit) and 3 samples with Maxima H Minus First Strand cDNA Synthesis Kit (ThermoFisher Scientific, https://www.thermofisher.com). To exclude the possibility that a high number of cycles could exacerbate cross-contamination of samples with low viral load, we ran either 18 or 24 cycles for the multiplex PCR step of library amplification. We also sent residual portions of 12 nasopharyngeal swab specimens to the New York State Department of Health (Albany, NY, USA) for comparative sequencing with a different platform and chemistry. The Department of Health performed RNA extraction with an easyMAG (bioMérieux, https://www.biomerieux.com) and library preparation and sequencing with an Ion Chef and Ion S5 XL System, using the Ion AmpliSeq SARS-CoV-2 Insight Research Assay with 27 cycles (ThermoFisher Scientific). Repeating the library preparations and resequencing by different methods produced sequences with no P681R calls, except for 2 samples that showed P681R with 24 PCR cycles and P681H with 18 cycles (Table), indicating that a high number of PCR cycles can introduce false mutation calls. Our combined experiments also confirmed that these were not errors induced by reverse transcriptase during the cDNA synthesis step.

**Table:** Results of repeated library preparations and sequencing of 25 random SARS-CoV-2 samples with Omicron variant and mutation previously assigned as P681R, including an alternative chemistry and sequencing platform*

Location		NYULH Genome Technology Center				NYSDOH
Protocol		xGen SARS-CoV-2 Amp Panel 96rxn†				AmpliSeq SARS-CoV-2 Insight‡
Reverse transcriptase	SuperScript IV		Maxima H Minus			SuperScript VILO cDNA synthesis kit§	
PCR cycles, no. P681R calls						
		18, 0		18, 0		27, 0
		24, 1		24, 1		NA

*NA, not applicable; NYSDOH, New York State Department of Health, Albany, NY, USA, which performed ThermoFisher sequencing chemistry on 12 samples; NYULH, NYU Langone Health, New York, NY, USA, which performed Illumina sequencing chemistry on 13 different samples; SARS-CoV-2, severe acute respiratory syndrome coronavirus 2. †Integrated DNA Technologies (https://eu.idtdna.com). ‡ThermoFisher Scientific (https://www.thermofisher.com). §Invitrogen (https://www.thermofisher.com).

We then reprocessed the remaining 61 samples with P681R, using the xGen kit with 18 cycles of amplification. A total of 59 samples showed P681H on this repeated testing; only 2 still showed P681R, 1 at 0.52 frequency (down from 0.98) and 1 at 0.94, exactly as the previous sequence, suggesting a true P681R call, possibly co-infection with Delta, because the C_t_ for this sample was low (C_t_ = 17). We performed an additional RNA extraction, library preparation, and sequencing for this sample; the P681R persisted at frequency 0.84, suggesting that this sample represents co-infection.

We conclude that the foremost reason for detecting P681R in our Omicron samples was contamination with Delta amplicons and artifactual mixed base pair calls, resulting from preferential coverage of that specific position and amplicon in the context of Delta but not Omicron. Although non–amplicon-based approaches such as capture-hybridization libraries using SARS-CoV-2 baits generally lead to more even coverage, and amplicon-based methods are known to result in dropouts because of new mutations in different variants, amplicon methods have been widely adopted by genomic surveillance laboratories under pressure for faster turnaround and high volumes, especially during large waves of infection. We urge laboratories to confirm unusual mutation findings by repeating libraries and sequencing or by using alternative protocols, or both, to avoid artifacts and ensure accurate sequences in databases such as GISAID, which are used by the global scientific community.
